# Effectiveness of Simulation-Based, Peer Learning Intervention in Continuing Nursing Education: An Explorative Study

**DOI:** 10.7759/cureus.62613

**Published:** 2024-06-18

**Authors:** Pecy M Paul, Sanchosekar M, Seetu Palo, Edward M Priyanka, Divya R Nair, Sathiya R

**Affiliations:** 1 Nursing, All India Institute of Medical Sciences, Hyderabad, IND; 2 Pathology and Laboratory Medicine, All India Institute of Medical Sciences, Hyderabad, IND

**Keywords:** in-service training, simulation, peer-learning, nursing, education

## Abstract

Background: Despite the vast research by nursing professionals on various methods of nursing education, little research has been conducted exploring the efficacy of peer learning as a teaching-learning tool amongst registered nurses. Hence, this study was conducted among in-service nursing officers to evaluate the usefulness of simulation-based peer learning sessions as an educational tool for capacity building.

Material and methods: Using a pre-test and post-test design, the study was conducted among 150 in-service nurses at a tertiary care hospital. Five structured simulation-based, peer learning modules were designed. The nurses were divided into five groups using random and purposive sampling. Each group attended one session of the peer learning module on advanced nursing care by simulated clinical and nursing care 'demonstrate, observe, assist, and perform' (DOAP) activity. Pre-test, post-test, and retention tests (after two months) were conducted, and the results were compared.

Results: There was a significant increase in mean knowledge (p-value < 0.05) in the post-test after all five sessions, which shows the effectiveness of such peer learning sessions in improving the baseline. There was a decline in mean scores in the retention test compared to that of the post-test, which was statistically significant in only the group of learners participating in the first session.

Conclusion: The study provides substantial evidence that simulation-based peer learning is an effective tool for continuing nursing education, and it can be used as a valuable tool to reduce the documented theory-practice gap.

## Introduction

In the field of nursing, ongoing professional development and continuous learning are critical to ensuring the delivery of high-quality patient care. In-service education plays a vital role in equipping nurses with updated knowledge and skills to meet the ever-evolving demands of the healthcare landscape. While traditional teaching methods have long been employed in nursing education, peer learning sessions have emerged as a promising approach to promote active learning, collaboration, and professional growth among nurses. Stone et al. systematically reviewed the utility of peer learning in undergraduate nursing education and concluded that peer learning is an effective student-centric teaching-learning methodology that aids students in becoming more proficient communicators and critical thinkers [1]. Simulation-based learning, yet another novel approach, has become a prominent educational method in nursing education, providing learners with realistic experiences in a safe and controlled environment. While many investigators have demonstrated simulation-based peer learning as an effective tool in training nursing students [1,2], there is a paucity of studies exploring its utility as in-service nursing education or continued nursing education. Hence, this study was conducted among in-service nursing officers to evaluate the effectiveness of simulation-based peer learning sessions as an educational tool for capacity building of in-service nursing professionals.

## Materials and methods

This study was conducted among in-service nursing officers at a tertiary care hospital and teaching institute. The recruitment of participants involved reaching out to potential nursing officers through emails and WhatsApp group messages. Consenting individuals were then invited to attend an informal briefing session about the study design of five proposed peer learning activities. Random and purposive sampling was done from the attendees, resulting in the enrolment of 30 nursing officers as participants/learners and five nursing officers with relevant work experience as facilitators for each peer learning session. Hence, the study sample comprised 150 nursing officers as participants. By incorporating random sampling of participants, selection bias was eliminated, thereby enhancing the validity of the study results. Purposive sampling was used to select nursing officers with relevant work experience as facilitators. This targeted approach ensured that the facilitators had the necessary skills and expertise to effectively guide the peer learning sessions, thereby enhancing the quality and effectiveness of the learning activities.

Subsequently, five structured, simulation-based, peer learning modules were designed by the nurses who were recruited to act as facilitators for their peers. These modules focused on advanced nursing care, specifically covering topics such as normal labor and new-born care, neonatal resuscitation program, renal replacement therapy (hemodialysis), handling of essential equipment in critical care (ventilators, defibrillator, arterial blood gas machine, syringe pump, and infusion pump) and best practices in phlebotomy and intravenous cannulation. To ensure the quality of these peer-assisted simulation-based interventions, each group of facilitators was guided by two expert doctors (acting as mentors) to develop these modules by adaptive mentorship model, that is, the mentors and facilitators collaboratively tailored the peer learning modules to the specific needs and skill levels of the participant groups [3].

Each peer learning session had a planned duration of three hours and was structured more or less similarly to maintain uniformity. At the start of each session, the participants were briefed about the session’s specific learning objectives and were directed to attempt the pre-test questionnaire. A score of ‘1’ was awarded for each correct response and ‘0’ for unanswered/incorrect responses. After collecting the pre-test responses, interactive didactic lectures, utilizing various audio-visual aids, were delivered by the facilitators to prime the participants on the topic. After a brief break of five minutes, simulated clinical and nursing care 'demonstrate, observe, assist and perform' (DOAP) activities were conducted with Peyton's four-step approach [4]. Simulations used were role-plays with standardized patients (for all five sessions); actual equipment (for the session on essential equipment in critical care); high-fidelity mannequin, namely the SimMom (for normal labor and new-born care); low-fidelity mannequins (for sessions on neonatal resuscitation program and renal replacement therapy); and task trainers (for the session on best practices in phlebotomy and intravenous cannulation).

**Figure 1:**
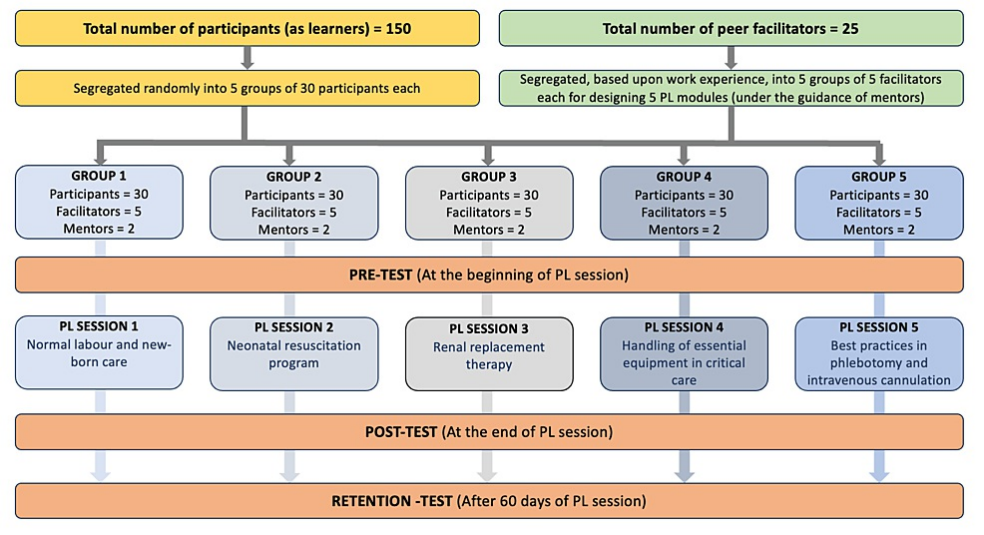
Overall framework of study design and methodology PL: Peer learning

**Figure 2:**
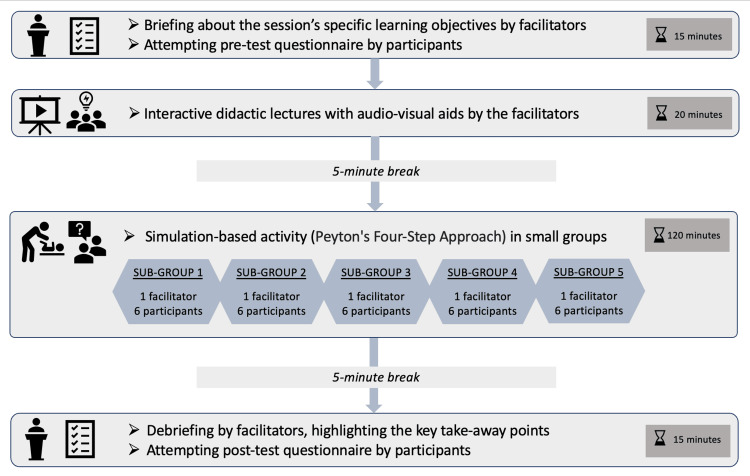
Detailed framework of a peer learning session

This simulation-based DOAP activity was conducted in five small groups, each group consisting of six participants and one facilitator, allowing sufficient time for open discussions, doubt clarification, and constructive feedback. In these peer learning sessions, the nurses who were acting as teachers/facilitators were proficient in the concerned topics and were able to interact in a more approachable manner with the learners. The mentors ensured the credibility, appropriateness, and quality of these teaching modules and also played a supervisory role. Just before the end, one of the facilitators, delivered a quick debriefing session, highlighting the key take-away points, and the participants were asked to answer the same questionnaire once again as post-test. A retention test was undertaken exactly two months after the initial peer learning session, wherein the same set of questionnaires was administered to the same set of participants to evaluate the effectiveness of these sessions (See Appendix A). Statistical analysis was performed using the Jamovi software version 2.5.5. Data points were analyzed using descriptive statistics (mean, median). A p-value less than 0.05 was considered to be statistically significant. The study framework is illustrated in Figures 1-2. 

This peer learning activity was conducted as a part of *International Nurses Day 2023 *and all the administrative approvals were taken. However, it was not reviewed by the Institutional Review Board (IRB) as it was initially not planned as a research activity.

## Results

**Figure 3:**
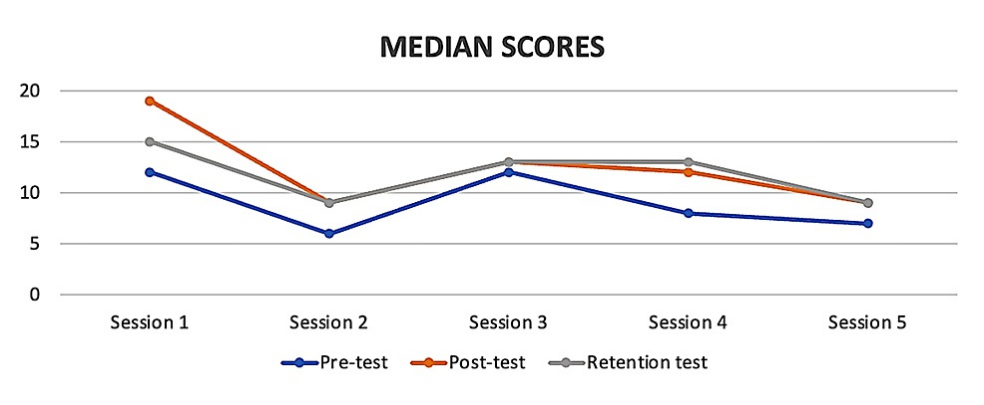
Median scores obtained by nurses in the pre-test, post-test, and retention test of each peer learning session

The basic demographic characteristics of the participants/learners are presented in Table 1. The average age of participants was approximately 29 years across all the five sessions. The age range showed slight variation, with the broadest range observed in peer learning session 3 (23 to 42 years). Females constituted the majority of participants in every session. Work experience differed among the sessions, spanning from 0 to 14 years. Table 2 and Figure 3 illustrate participants' mean and median test scores in pre-test, post-test, and retention tests in all five peer learning sessions, respectively. For each peer learning session, the mean and median scores of the post-test and retention test were higher than that of the pre-test, indicating improvement of baseline knowledge after each session. Although there was a decrease in mean scores from the post-test to the retention test, this decline was statistically significant only for the first session. In the other four sessions, the differences between post-test and retention test scores were not statistically significant, suggesting that while some knowledge was retained, there was no significant drop or further improvement. Typically, the mean retention test scores were lower than the mean post-test scores but remained higher than the mean pre-test scores.

**Table 1:** Demographics of the participant nurses PL: Peer learning; M:F: Male: female

Demographics	PL Session 1	PL Session 2	PL Session 3	PL Session 4	PL Session 5
No. of participants	30	30	30	30	30
Mean age (in years)	29.1	28.43	29.9	28.43	30.06
Age range (in years)	22 – 35	24 – 34	23 – 42	24 – 40	24 – 38
M:F	2:28	9:21	5:25	10:20	5:25
Work experience (in years)	0 – 11	0 - 10	0 - 14	0 - 12	0 - 11

**Table 2:** Comparison of mean knowledge scores of nurses in the pre-test, post-test, and retention test of each peer learning session

PL sessions	Maximum score	No. of participants	Mean score (SD)			p-value		
			Pre-test	Post-test	Retention test	Pre-test vs. Post-test	Pre-test vs. Retention test	Post-test vs. Retention test
1	20	20	12.55 (3.14)	18.40 (1.54)	15.80 (2.26)	<0.001	<0.001	<0.001
2	10	27	6.52 (1.97)	8.70 (1.33)	8.30 (1.73)	<0.001	<0.001	0.234
3	15	22	11.45 (1.66)	13.23 (1.23)	12.95 (1.70)	0.002	0.001	0.553
4	15	21	8.38 (2.52)	11.52 (3.03)	11.81 (2.84)	<0.001	<0.001	0.642
5	10	21	6.57 (0.98)	8.81 (1.25)	8.71 (1.23)	<0.001	<0.001	0.754

## Discussion

Continuing nursing education consists of organized learning opportunities that enhance nurses' attitudes, knowledge, and skills on newer treatment trends and, as a result, positively strengthen the quality of patient care. But the question is which method of teaching will achieve the maximum desired outcome. Attending conferences, seminars, and workshops are the conventional and prevalent platforms for ongoing nursing education and professional growth, but may not be accessible and feasible to all nurses. Eslamian et al. delved into learners-related, teachers-related, and logistics-related barriers in continuing nursing education and have proposed the need to revisit and revise the educational process to adequately meet nurses' needs [5]. This calls for exploring the effectiveness and feasibility of novel learner-centric teaching methodologies, such as peer learning. Although nurses keep imbibing knowledge and skills on a day-to-day basis from doctors, seniors, and fellow nurses, formal peer learning activities have not yet been established as an efficient tool for continuing education for practicing nurses.

Peer learning is defined by Topping as "the acquisition of knowledge and skill through active helping and supporting among status equals or matched companions" [6]. Boud defined it in more simplified terms as "students learning from and with each other in both formal and informal ways" [6]. Currently, a myriad of terminologies, such as cooperative learning, peer tutoring, peer mentoring, near-peer teaching, peer coaching, team learning, etc., are used to acknowledge the concept of peer learning [1]. Peer learning sessions can be structured as per needs and objectives. It can be designed in a one-to-one, one-to-many, or many-to-many format and can be formal or informal [7]. In the current study, the many-to-many (many facilitators and many learners/participants) model was used, as we think that this is the best feasible model for registered nurses.

In our questionnaire-based study, there was a significant increase in mean knowledge (p-value <0.05) in the post-test after all five sessions, which shows the effectiveness of such peer learning sessions in improvising baseline knowledge. Similar usefulness of peer learning in nursing students’ education has been demonstrated by various investigators [8,9,10]. Parmar et al. compared peer learning versus conventional methods regarding antenatal assessment among nursing students and observed both to be equally effective in improving knowledge and skill levels [11]. In another study, peer learning was found to be as efficient as self-directed learning in enhancing nursing students’ learning and ability to interpret electrocardiograms [12]. Nelwati et al. conducted a quasi-experimental study to evaluate the effect of peer learning on professional competence development among undergraduate nursing students and obtained encouraging results [13]. Apart from augmenting academic knowledge and refining clinical competence, utilizing peer learning methods during the educational process yields numerous added advantages, such as enhancing social skills, improving communication skills, developing empathy, and boosting self-confidence [14]. Many investigators have asserted that during the initial stages of clinical placement, students typically place a high value on their relationships with near-peers, and by offering mutual support, peers can assist each other in navigating the new environment, identify areas of knowledge and skill gaps, and help each other in obtaining new learning experiences [15]. The same can be extrapolated to registered nurses as well, wherein peers and near-peers can support one another and provide a conducive environment for active learning and adaptation in the new workplace.

**Figure 4:**
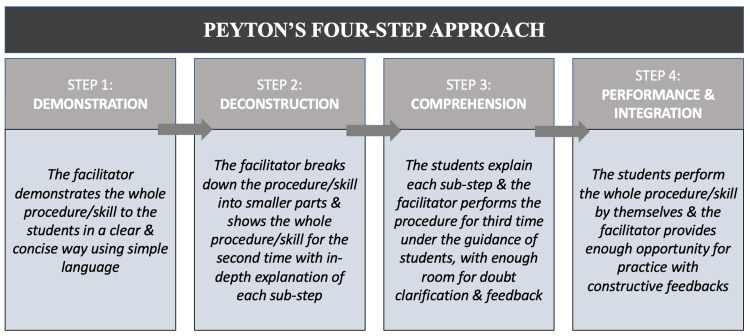
Gist of Peyton’s four-step approach

For obtaining optimum results, it is imperative to design peer learning sessions in a very thoughtful manner, keeping in consideration the learners’ needs, desired learning outcomes, available logistics, time constraints, etc. The use of appropriate pedagogical methods plays a pivotal role. Putri et al. carried out a post-test-only-control-group design on 31 nursing students with problem-based, peer learning as an intervention and found it to directly improve students’ communication skills, nursing care, and overall professional approach [9]. Golaki et al. used the flipped classroom approach for near-peer learning on patient safety knowledge retention in nursing students and found it effective in improvising baseline knowledge [16]. Other teaching and learning methods can be case-based learning, think-pair-share, the jigsaw method, Socratic seminars, panel discussions, presentations, etc. We used a simulation-based method for delivering the module content, as it is a well-documented and established method to promote clinical skills, critical thinking, self-reliance, and leadership qualities [17,18]. Simulation-based learning is an instructional approach that replicates real-world clinical scenarios, providing learners with the opportunity to practice the procedure repeatedly and experience hands-on training in a secure and conducive environment wherein making errors is permissible. A variety of simulation methods can be used in nursing education, such as high-fidelity mannequins, low-fidelity mannequins, partial task simulators, role-plays with standardized patients, virtual reality applications, etc. [19]. We devised a robust study framework by incorporating Peyton's four-step approach and using a combination of simulations to create a more realistic simulation and maximize the benefits of simulation-based learning (Figure 4). Peyton’s four-step approach is a widely recognized method for teaching adult learners and involves four steps: (i) demonstration; (ii) deconstruction; (iii) comprehension; and (iv) performance and integration [4]. Overall, it is a very effective method that emphasizes clearing concepts by observation and ‘do-it-yourself’ experience and also provides opportunities for practice and feedback.

In this study, although there was a significant increase in knowledge scores in the post-test and retention test as compared to baseline knowledge scores, there was a decline in knowledge scores in the retention test compared to that of the post-test, which highlights the fact that knowledge retention is dependent upon a multitude of factors. The plausible reasons for knowledge decay can be attributed to the learner's interest in the topic of the peer learning module, prior work experience, and current work profile. For example, a participant in a session on maternal care did not get the opportunity to practice (‘on-the-job’ training) what was learned from the simulation-based, peer learning session. A similar phenomenon was reported by Srivilaithon et al., who studied the degree of retention of basic life support knowledge and skills amongst second-year medical undergraduate students [20]. They reported that the knowledge levels and some skill components declined in the retention test but were still higher than those of the pre-test. Srivilaithon et al. and others have also proposed the conduct of periodic reinforcement training modules for facilitating adequate retention of knowledge and skills [20,21,22]. Although ideal, carrying out periodic training might not be a feasible option in all instances, and therefore importance should be given to ‘on-the-job’ training. Also, handouts and video recordings of the sessions can be provided to the nurses for self-directed learning.

On the positive side, DOAP stations with Peyton’s four-step approach followed by open discussion paved the way for the participants to clarify their doubts from colleagues, facilitators, and mentors, leading to an engaging and enriching learning experience. The use of various modes of simulation made the sessions more interesting and provided hands-on experience. Hence, this study effectively harnessed the potential of two powerful pedagogical methods, namely, peer learning and simulation-based learning. Additionally, the interprofessional education, i.e., the mentors (doctors in the particular field) in these peer learning sessions, helped bridge the gap between the expectations of the doctors and the practice of the nurses. This shared learning can be introduced at the undergraduate level itself, which will lead to a reduction of negative stereotypes and provide synergistic clinical and nursing care to patients, and registered nurses can utilize greater clinical thinking when assessing patients.

However, there are a few limitations to the study, and few lessons were learned. From the study viewpoint, first, a relatively small sample size and purposive sampling technique were used in the present study. Hence, it cannot be generalized for other settings. Second, this was a quantitative study. A mixed-method study design with a qualitative component would have helped us to gather a better understanding of mentors', facilitators', and learners' perspectives. In retrospect, we feel that in addition to using self-reporting questionnaires for knowledge assessment, objective structured clinical examination (OSCE) stations could have been added for the evaluation of clinical competencies achieved after the sessions. We also learned that rather than randomly grouping the learners, need-based and interest-based grouping would be more beneficial for such peer learning sessions. Also, an adaptive mentorship model can be followed while framing peer learning sessions, where the mentors and facilitators can collaboratively tailor the learning modules to the specific needs and skill levels of the participant groups. Another area where improvisation can be made is the mode of debriefing. Facilitator-led verbal debriefing was done at the end of each peer learning session. Niu et al. conducted a systematic review and meta-analysis of debriefing methods in nursing education and concluded that video-assisted debriefing and structured debriefing methods are associated with better learning outcomes in contrast to usual verbal debriefing [23].

## Conclusions

This study provides strong evidence that simulation-based peer learning can serve as an effective teaching-learning method for ongoing nursing education. The findings of the study indicate that the peer learning modules, designed and facilitated by experienced nurses with guidance from mentors, can effectively improve baseline knowledge and demonstrate sustained retention over time. The innovative use of simulations, combined with Peyton’s four-step approach and a structured debriefing process in the peer learning sessions, promotes active learning, collaboration, and skill development among nursing personnel. This study also provides preliminary insights on how to conduct peer learning sessions and provides a model that can be adapted and replicated in other institutions. Overall, this study contributes to the growing body of evidence supporting simulation-based peer learning as a valuable educational tool for in-service nursing professionals. By fostering continuous learning and professional development, such initiatives can ultimately lead to improved patient care.

## Appendices

Appendix A

Below is the set of questionnaires used for knowledge assessment in each of the peer learning sessions.

**Table 3:** Performance of participants in the pre-test, post-test, and retention test of peer learning session 1 on ‘Normal Labour and Newborn Care’ The correct answers to the questions are highlighted in bold. APGAR:  Appearance, pulse, grimace, activity, and respiration; BPM: Beats per minute

No.	Questions	No. of participants (%) who answered correctly		
		Pre-test (n=26)	Post-test (n=24)	Retention test (n=27)
1	Abbreviation of RMC:	14 (53.8%)	23 (95.8%)	26 (96.3%)
	(a) Related maternity care			
	(b) Responsible maternal care			
	(c) Respectful maternity care			
	(d) Religious maternal care			
2	Shouting and scolding using abusive language is an example of:	19 (73.1%)	22 (91.7%)	24 (88.9%)
	(a) Negligence			
	(b) Non-dignified care			
	(c) Non-consented care			
	(d) Discrimination			
3	Calculate the expected date of delivery (EDD) using Nagele's rule, for the last menstrual period October 19, 2023:	24 (92.3%)	24 (100%)	26 (96.3%)
	(a) July 12, 2024			
	(b) July 26, 2023			
	(c) July 26, 2024			
	(d) August 12, 2024			
4	Lateral grip is otherwise called as:	12 (46.2%)	21 (87.5%)	20 (74.1%)
	(a) Pelvic grip			
	(b) Pawlick grip			
	(c) Umbilical grip			
	(d) Fundal grip			
5	Variable deceleration is seen in:	15 (57.7%)	19 (79.2%)	18 (66.7%)
	(a) Head compression			
	(b) Cord compression			
	(c) Placental insufficiency			
	(d) Pre-term baby			
6	Normal heart rate variability ranges from (BPM):	15 (57.7%)	23 (95.8%)	22 (81.5%)
	(a) 0-10			
	(b) 5-25			
	(c) 10-30			
	(d) 0-50			
7	In the mechanism of labor, the twist in the fetus which is corrected by a slight untwisting movement is called:	17 (65.4%)	24 (100%)	23 (85.2%)
	(a) Internal rotation			
	(b) Restitution			
	(c) External rotation			
	(d) Lateral flexion			
8	The widest diameter of the fetal head is:	8 (30.8%)	15 (62.5%)	6 (22.2%)
	(a) Biparietal diameter			
	(b) Suboccipito-bregmatic			
	(c) Bitemporal diameter			
	(d) Occipito-frontal			
9	Recommended or preferred drug for prevention of PPH during active management of 3rd stage of labor is:	11 (42.3%)	22 (91.7%)	22 (81.5%)
	(a) Inj. oxytocin 10 U			
	(b) Tab. Misoprostol 200 mcg			
	(c) Inj. Methergine 0.2 mg			
	(d) Inj. Carboprost 250 mcg			
10	Duration of the post-partum period is:	24 (92.3%)	24 (100%)	26 (96.3%)
	(a) Up to 6 weeks post-delivery			
	(b) Up to 2 weeks post-delivery			
	(c) Up to 4 weeks post-delivery			
	(d) Up to 12 weeks post-delivery			
11	A normally involuting uterus will be:	5 (19.2%)	19 (79.2%)	13 (48.1%)
	(a) Soft and tender			
	(b) Soft and non-tender			
	(c) Firm and tender			
	(d) Firm and non-tender			
12	Phases of psychological changes during the post-partum period are:	23 (88.5%)	23 (95.8%)	24 (88.9%)
	(a) Taking in			
	(b) Taking hold			
	(c) Letting go			
	(d) All of the above			
13	Which of the following is not included in active management of 3rd stage of labor?	10 (38.5%)	21 (87.5%)	16 (59.3%)
	(a) Administration of uterotonic within 1 min of delivery			
	(b) Early cord clamping			
	(c) Gentle massage of the uterus			
	(d) Controlled cord traction			
14	Which vital sign is included in the APGAR score?	23 (88.5%)	24 (100%)	25 (96.6%)
	(a) Temperature			
	(b) Heart rate			
	(c) Meconium-stained			
	(d) Edema			
15	A nurse in the delivery room is assisting with the delivery of a newborn. After the delivery, the nurse prepares to prevent heat loss in the newborn resulting from evaporation by:	20 (76.9%)	22 (91.7%)	20 (74.1%)
	(a) Warming the crib pad			
	(b) Turning on the overhead radiant warmer			
	(c) Closing the door			
	(d) Drying the infant in a warm blanket			
16	Immediate care of newborn means:	24 (92.3%)	24 (100%)	27 (100%)
	(a) Care of baby at birth in the labor room			
	(b) Care of baby in the postnatal ward			
	(c) Care of baby after discharge			
	(d) None of the above			
17	Indicator of referral in partograph except:	19 (73.1%)	21 (87.5%)	19 (70.4%)
	(a) Action line			
	(b) Meconium stain liquor			
	(c) Prolonged labor			
	(d) 4 cm dilatation			
18	Meconium-stained liquor is marked in partograph as:	21 (80.8%)	24 (100%)	24 (88.9%)
	(a) A			
	(b) C			
	(c) M			
	(d) L			
19	The temperature to be maintained in the labor room is (degree Celsius)	12 (46.2%)	21 (87.5%)	17 (63%)
	(a) 30-32			
	(b) 22-24			
	(c) 26-28			
	(d) 24-28			
20	Suboccipito-frontal diameter is:	8 (30.8%)	23 (95.8%)	16 (59.3%)
	(a) 9cm			
	(b) 10cm			
	(c) 12cm			
	(d) 13cm			

**Table 4:** Performance of participants in the pre-test, post-test, and retention test of peer learning session 2 on ‘Neonatal Resuscitation Program’ The correct answers to the questions are highlighted in bold. BPM: Beats per minute

No.	Questions	No. of participants (%) who answered correctly		
		Pre-test (n=30)	Post-test (n=32)	Retention test (n=27)
1	Normal neonatal heart rate is:	27 (90%)	31 (96.9%)	24 (88.9%)
	(a) 100 to 150 BPM			
	(b) 120 to 160 BPM			
	(c) 110 to 160 BPM			
	(d) 120 to 200 BPM			
2	Appropriate indication for starting chest compression in a neonates is:	16 (53.3%)	23 (71.9%)	17 (65.4%)
	(a) Heart rate <100 BPM			
	(b) Heart rate <150 BPM			
	(c) Heart rate <60 BPM			
	(d) Heart rate <80 BPM			
3	Adrenaline dosage in neonatal resuscitation is:	17 (56.7%)	26 (81.3%)	20 (74.1%)
	(a) 1:100			
	(b) 1:1000			
	(c) 1:10,000			
	(d) 1:100,000			
4	What is the preferred route of drug administration in a neonate, if the IV line is not accessible?	12 (40%)	31 (96.9%)	22 (81.5%)
	(a) Intramuscular			
	(b) Subcutaneous			
	(c) Intraosseous			
	(d) Both a & c			
5	Which of the following is the correct head position of a neonate while performing resuscitation?	21 (70%)	27 (84.4%)	26 (96.3%)
	(a) Flexion			
	(b) Slightly extended			
	(c) Hyper extended			
	(d) None of the above			
6	The compression-to-ventilation ratio for cardiopulmonary resuscitation (CPR) in a neonate is:	22 (73.3%)	28 (87.5%)	23 (85.2%)
	(a) 1 compression to 3 ventilations			
	(b) 2 compressions to 1 ventilation			
	(c) 3 compressions to 3 ventilations			
	(d) 3 compressions to 1 ventilation			
7	If the newborn's heart rate is still below 60 bpm despite ventilation and chest compressions, what is the most appropriate next step?	17 (56.7%)	27 (84.4%)	21 (77.8%)
	(a) Administer epinephrine			
	(b) Provide continuous positive airway pressure (CPAP)			
	(c) Check the baby for signs of pneumothorax			
	(d) Start dopamine			
8	The preferred technique for delivering chest compression in a neonate is:	17 (56.7%)	29 (90.6%)	25 (92.6%)
	(a) Two-thumb encircling hands technique			
	(b) Two-finger technique			
	(c) Chests thrusts			
	(d) None of the above			
9	For successful neonatal resuscitation, the following is/are needed:	23 (76.7%)	27 (84.4%)	26 (96.3%)
	(a) Anticipation			
	(b) Adequate preparation			
	(c) Skilled personnel			
	(d) Delayed initiation of support			
10	The following are true in relation to the initial steps of neonatal resuscitation:	16 (55.2%)	29 (90.6%)	20 (74.1%)
	(a) Provide warmth			
	(b) Tactile stimulation			
	(c) Endotracheal intubation			
	(d) Drying the baby			

**Table 5:** Performance of participants in the pre-test, post-test, and retention test of peer learning session 3 on ‘Renal Replacement Therapy: Hemodialysis’ The correct answers to the questions are highlighted in bold. Mg: Magnesium, Zn: Zinc, Na: Sodium, K+: Potassium

No.	Questions	No. of participants (%) who answered correctly		
		Pre-test (n=26)	Post-test (n=27)	Retention test (n=27)
1	Hemodialysis rids your body of harmful wastes. What else does hemodialysis remove?	16 (61.5%)	23 (85.2%)	20 (74.1%)
	(a) Extra water			
	(b) Extra protein			
	(c) Extra glucose			
	(d) Extra salt			
2	What is a common side effect of hemodialysis?	25 (96.2%)	27 (100%)	27 (100%)
	(a) Muscle cramps			
	(b) Dizziness			
	(c) Nausea			
	(d) All of the above			
3	What is the filter that acts as an artificial kidney called?	23 (88.5%)	27 (100%)	27 (100%)
	(a) Dialyzer			
	(b) Hemolyzer			
	(c) Nephrolyzer			
	(d) None of the above			
4	Golden access for treatment that is used in hemodialysis:	22 (84.6%)	23 (85.2%)	24 (88.9%)
	(a) AV graft			
	(b) AV fistula			
	(c) Tunneled catheter			
	(d) Non tunneled catheter			
5	What is the composition of the membrane used in dialysis?	17 (65.4%)	19 (70.4%)	19 (70.4%)
	(b) Polyethylene			
	(a) Chitin			
	(c) Polyvinyl chloride			
	(d) Cellulose			
6	Most common side effect of hemodialysis:	24 (92.3%)	27 (100%)	26 (96.3%)
	(a) Hypotension			
	(b) Hyperglycemia			
	(c) Hypo Magnesemia			
	(d) Hyperkalemia			
7	Different types of hemodialysis:	26 (100%)	27 (100%)	26 (96.3%)
	(a) Sled			
	(b) Hemodiafiltration (HDF)			
	(c) Continuous venovenous hemodialysis (CVVHD)			
	(d) All of the above			
8	Frequency of hemodialysis:	12 (46.2%)	25 (92.6%)	22 (81.5%)
	(a) 2 times a week			
	(b) 1 time a week			
	(c) 3 times a week			
	(d) 4 times a week			
9	Injections are administered:	18 (69.2%)	21 (77.8%)	21 (77.8%)
	(a) Before dialysis			
	(b) During dialysis			
	(c) After dialysis			
	(d) None of the above			
10	If air embolism is suspected during hemodialysis, what is the first action taken by the nurse?	16(61.5%)	20 (74.1%)	23 (85.2%)
	(a) Administer oxygen			
	(b) Left lateral position			
	(c) Clamp the line			
	(d) Document the findings			
11	Anticoagulant used in hemodialysis:	25 (96.2%)	26 (96.3%)	26 (96.3%)
	(a) Warfarin			
	(b) Heparin			
	(c) Streptokinase			
	(d) Aspirin			
12	Principles of hemodialysis:	20 (76.9%)	27 (100%)	25 (92.6%)
	(a) Diffusion			
	(b) Osmosis			
	(c) Ultrafiltration			
	(d) All of the above			
13	How does the diet differ for someone on hemodialysis from a patient on peritoneal dialysis?	12 (46.2%)	20 (74.1%)	12 (44.4%)
	(a) It requires more calories			
	(b) It requires more calcium			
	(c) It requires less protein			
	(d) None of the above			
14	The mineral that must be consumed in limited quantities by those undertaking hemodialysis:	17 (65.4%)	19 (70.4%)	21 (77.8%)
	(a) Mg			
	(b) Zn			
	(c) Na			
	(d) K+			
15	Apart from the conventional use, dialysis can also be used in scenarios of:	26 (100%)	27 (100%)	27 (100%)
	(a) Blood transfusions			
	(b) Acute poisoning			
	(c) Low blood pressure			
	(d) Extreme fever			

**Table 6:** Performance of participants in the pre-test, post-test, and retention test of peer learning session 4 on ‘Handling of Essential Equipments in Critical Care’ The correct answers to the questions are highlighted in bold. PEEP: Positive end-expiratory pressure, I:E: Inspiratory to expiratory, FiO2: Fraction of inspired oxygen, PO2: Partial pressure of oxygen, SPO2: Saturation of peripheral oxygen, CO2: Carbon dioxide, SIMV: Synchronized intermittent mandatory ventilation, ABG: Arterial blood gas

No.	Questions	No. of participants (%) who answered correctly		
		Pre-test (n=30)	Post-test (n=30)	Retention test (n=23)
1	______ is flow multiplied by time.	25 (83.3%)	27 (90%)	20 (87%)
	(a) Pressure			
	(b) Resistance			
	(c) Volume			
	(d) Flow			
2	Which one of the following modes of ventilation runs the risk of the patient getting respiratory alkalosis?	2 (6.7%)	14 (46.7%)	12 (52.2%)
	(a) Controlled mandatory ventilation			
	(b) Assist control mode			
	(c) Pressure control mode			
	(d) Synchronized intermittent mandatory ventilation (SIMV) mode			
3	The mode of ventilation that allows the patient to breathe spontaneously at his or her own respiratory rate and depth between the ventilator breath is _______:	20 (66.7%)	28 (93.3%)	21 (91.3%)
	(a) Pressure support mode			
	(b) Volume control mode			
	(c) Synchronized intermittent mandatory ventilation (SIMV) mode			
	(d) Pressure control mode			
4	Which of the following conditions require a higher PEEP to be applied in recruiting collapsed alveoli?	11 (36.7%)	13 (43.3%)	17 (73.9%)
	(a) Asthma			
	(b) Acute respiratory distress syndrome			
	(c) Emphysema			
	(d) Bronchiectasis			
5	Which one of the following is a risk of keeping high PEEP?	8 (26.7%)	15 (50%)	19 (82.6%)
	(a) Hypotension			
	(b) Hypertension			
	(c) Respiratory alkalosis			
	(d) Respiratory acidosis			
6	Once mechanical ventilation is established, which of the following suggests that an intraluminal mass or bronchospasm is present?	18 (60%)	24 (80%)	17 (73.9%)
	(a) Low minute ventilation			
	(b) Elevated resistive pressure			
	(c) Inappropriate tidal volume for the lung			
	(d) Increased elastic pressure			
7	The amount of oxygen dissolved in plasma is _____:	20 (66.7%)	27 (90%)	18 (78.3%)
	(a) FiO2			
	(b) PO2			
	(c) SPO2			
	(d) CO2			
8	________ pressure is measured at the end of inspiration with an inspiratory hold maneuver.	18 (60%)	22 (73.3%)	21(91.3%)
	(a) Peak pressure			
	(b) Plateau pressure			
	(c) Airway pressure			
	(d) Inspiratory pressure			
9	Which one of the following modes of ventilation 'locks out' the patient's efforts to breathe?	17 (56.7%)	23 (76.7%)	19(82.6%)
	(a) Controlled mandatory ventilation			
	(b) Pressure support ventilation			
	(c) Assist control ventilation			
	(d) SIMV Mode			
10	What are the ventilator parameters adjusted to maintain the optimum minute ventilation?	11(36.7%)	18 (60 %)	15(65.2%)
	(a) FIO2 and PEEP			
	(b) Tidal volume and I:E ratio			
	(c) Tidal volume and respiratory rate			
	(d) Trigger and flow			
11	After Allen's test, if the color of the palm returns to normal within 6 to 7 seconds it is said to be _____.	18 (60%)	27 (90%)	18 (78.3%)
	(a) Normal			
	(b) Abnormal			
	(c) Neutral			
	(d) None of the above			
12	To prevent backflow of blood from the arterial line, the pressure bag is inflated at _____mmHg.	13 (43.3%)	18 (60%)	13 (56.5%)
	(a) 200			
	(b) 250			
	(c) 300			
	(d) 350			
13	Which of the following statement is correct, after taking the ABG sample?	16 (53.3%)	30 (100%)	23 (100%)
	(a) Expel the air bubbles			
	(b) Cap the syringe			
	(c) Roll the specimen between the hands			
	(d) All of the above			
14	The placement of the defibrillator pad for adults is in the ________.	19 (63.3%)	27 (90%)	20 (87%)
	(a) 3^rd^ Intercostal space to the right of the sternum and 5^th^ intercostal space on the left mid-axillary line			
	(b) 3^rd^ Intercostal space to the left of the sternum and 5^th^ intercostal space on the right mid-axillary line			
	(c) 5^th^ Intercostal space to the left of the sternum and 3^rd^ intercostal space on the right mid-axillary line			
	(d) 5^th^ intercostal space to the right of the sternum and 3rd intercostal space on the left mid-axillary line			
15	The recommended shock energy for an adult patient in a biphasic defibrillator is:	20(66.7%)	28 (93.3%)	21 (91.3%)
	(a) 40 to 120 joules			
	(b) 120 to 200 joules			
	(c) 200 to 280 joules			
	(d) 280 to 360 joules			

**Table 7:** Performance of participants in the pre-test, post-test, and retention test of peer learning session 5 on ‘Best Practices in Phlebotomy and Intravenous Cannulation’ The correct answers to the questions are highlighted in bold. BUN: Blood urea nitrogen

No.	Questions	No. of participants (%) who answered correctly		
		Pre-test (n=26)	Post-test (n=28)	Retention test (n=28)
1	Angle for venipuncture:	21 (80.8%)	27 (94.4%)	27 (96.4%)
	(a) 15-30			
	(b) 45			
	(c) 90			
	(d) 60			
2	If a tourniquet is applied for longer than 3 minutes, which of the following analyte results will most likely elevate?	3 (11.5%)	14 (5%)	21 (75%)
	(a) Glucose			
	(b) Protein			
	(c) Potassium			
	(d) All of the above			
3	Grey-topped tubes contain a chemical that:	23 (88.5%)	22 (78.6%)	22 (78.6%)
	(a) Stops the action of insulin on glucose			
	(b) Prevents blood from clotting			
	(c) Clots the blood faster			
	(d) Increases blood pressure			
4	What color-topped tube would you use to draw BUN and creatinine?	12 (46.2%)	25 (89.3%)	26 (92.9%)
	(a) Grey			
	(b) Blue			
	(c) Red			
	(d) None of the above			
5	The light blue tube contains sodium citrate and is used to collect blood specimens for:	25 (96.2%)	27 (96.4%)	27 (96.4%)
	(a) Complete blood picture			
	(b) Blood culture			
	(c) Blood sugar			
	(d) Prothrombin time			
6	The first choice of vein for cannulation will be:	25 (96.2%)	28 (100%)	27 (96.4%)
	(a) Scalp vein			
	(b) Vein of the left lower extremity			
	(c) Veins of the forearm			
	(d) Vein present over a joint			
7	Based on universal infection control guidelines, an IV cannula should be changed within:	6 (23.1%)	23 (82.1%)	17 (60.7%)
	(a) 24-48 hours			
	(b) 48-72 hours			
	(c) 72-96 hours			
	(d) Should never be changed			
8	Hand hygiene:	24 (92.3%)	25 (89.3%)	25 (89.3%)
	(a) Should be done only if your hands are visibly soiled			
	(b) Is important and must be done			
	(c) Should be done if you have time			
	(d) Other			
9	Which of the following can be prevented if asepsis is maintained?	26 (100%)	27 (96.4%)	26 (92.9%)
	(a) Hematoma			
	(b) Air embolism			
	(c) Phlebitis			
	(d) Extravasation			
10	When should documentation of the IV cannulation procedure be done?	24 (92.3%)	28 (100%)	26 (92.9%)
	(a) As soon as the patient comes			
	(b) After the procedure is done			
	(c) Can be done at any time			
	(d) documentation is not necessary			
